# Two novel *AGXT* mutations identified in primary hyperoxaluria type-1 and distinct morphological and structural difference in kidney stones

**DOI:** 10.1038/srep33652

**Published:** 2016-09-20

**Authors:** Cui Wang, Jingru Lu, Yanhua Lang, Ting Liu, Xiaoling Wang, Xiangzhong Zhao, Leping Shao

**Affiliations:** 1 Department of Nephrology, the Affiliated Hospital of Qingdao University, 16 Jiangsu Road, Qingdao, 266003, China; 2 Central Laboratory, the Affiliated Hospital of Qingdao University, 16 Jiangsu Road, Qingdao 266003, China

## Abstract

Primary hyperoxaluria type 1 (PH1) is a rare genetic disease characterized by excessive oxalate accumulation in plasma and urine, resulting in various phenotypes because of allelic and clinical heterogeneity. This study aimed to detect disease-associated genetic mutations in three PH1 patients in a Chinese family. All *AGXT* exons and 3 common polymorphisms which might synergistically interact with mutations, including P11L, I340 M and IVSI+74 bp were analyzed by direct sequencing in all family members. It demonstrated that in each of three patients, a previously reported nonsense mutation p.R333^*^ was in cis with a novel missense mutation p.M49L in the minor allele characterized by the polymorphism of 74-bp duplication in intron 1, while the other novel missense mutation p.N72I was in trans with both p.R333^*^ and P.M49L in the major allele. Kidney stones from two sibling patients were also observed though stereomicroscopic examination and scanning electron microscopy. Distinct morphological and inner-structure differences in calculi were noticed, suggesting clinical heterozygosity of PH1 to a certain extent. In brief, two novel missense mutations were identified probably in association with PH1, a finding which should provide an accurate tool for prenatal diagnosis, genetic counseling and screening for potential presymptomatic individuals.

The primary hyperoxalurias (PHs) are rare inherited disorders of glyoxylate metabolism, in which specific enzyme deficiencies would result in excessive endogenous production of oxalate. Accumulated oxalate combines with calcium to form insoluble calcium oxalate (CaOx) crystals that are likely to deposite in kidneys and other potential target organs, such as eye, retina, heart or bone, leading to renal failure and functional impairments of other organs^1^. There are three forms of PHs identified to date. The most common and the most severe one is PH1 accounting for approximately 80% of all patients, caused by insufficiency or loss of activity of liver-specific peroxisomal alanine-glyoxylate aminotransferase (AGT)^2^, which is a compact homodimer and belongs to the aspartate aminotransferase family of pyridoxal-phosphate (PLP) dependent enzymes^3^. Primary hyperoxaluria type 2 (PH2), a less severe disease than PH1, is associated with deficiencies in the ubiquitously expressed gloxylate reductase (GR)/hydroxypyruvate reductase (HPR)^4^ and is responsible for about 10% of genetically characterized PH cases^5^. Primary hyperoxaluria type 3 (PH3), which represents the least severe PH form with good preservation of kidney function in most patients, is induced by gene mutations relevant with 4-hydroxy-2-oxoglutarate aldolase 1 (HOGA1)^6^.

The single copy of AGXT gene located on chromosome 2p37.3 is comprised of 11 exons spanning about 10 kb as the coding sequence of AGT^7^. Thus far, over 190 disease-causing mutations have been identified throughout *AGXT*; the three most recurrent mutations worldwide, p.G170R, c.33dupC, and p.I244T, account for approximately 30%, 11%, and 6% of *AGXT* mutant alleles, respectively^5^,^8^, suggesting a possibility of mutation “hot spots” in exon 4,1 and 7 of *AGXT*, severally. As we all know that *AGXT* is present in humans as two polymorphic variants, the most common allele “major allele” and the less frequent one “minor allele”. Minor alleles differ from major alleles by three polymorphisms: a 74-bp duplication in intron 1 and two mutations leading to the P11L and I340M amino acid substitutions, respectively^8^,^9^,^10^. Moreover, some mutations co-segregate and functionally interact with the minor allele polymorphisms, unmasking more potential molecular mechanism of PH1^11^,^12^,^13^. Owing to complicated genotypic and phenotypic heterogeneity, clinical spectrum of PH1 ranges from occasional stone formation to infantile nephrocalcinosis even end stage renal disease (ESRD)^14^, which may cause unavoidable underdiagnosis or delayed diagnosis of PH1. In the past, an accurate diagnosis of PH1 usually relies on a needle liver biopsy; recently, a molecular genetic detection has been considered as an efficient alternative and non-invasive approach to confirm PH1 patients^15^. In addition, as an important evidence of PH1, specific morphological and compositional features of kidney stones, might prompt early diagnosis among suspected patients^16^. Once a PH1 diagnosis is established, conservative treatment measures, like hyperhydration, urine alkalization or pyridoxine should be initiated to prevent precipitation of calcium oxalate and to slow progression to ESRD^17^,^18^.

The PH1 prevalence and incidence remains undetermined in China, and only few sporadic cases have been reported so far. Here, we performed clinical and genetic investigation of a large Chinese PH1 pedigree (Fig. 1), in which seven out of twenty members were heterozygotes and three siblings were PH1 patients with compound heterozygous variants. Two novel *AGXT* missense mutations, and distinct morphology and inner-structure difference of kidney stones were found in this kindred, meanwhile, the possible underlying pathogenic mechanisms at molecular and clinical levels were described.

## Results

### Clinical course of patients

Basic information and biochemical results of three patients in this family were shown in Table 1. The proband IIa with a long medical history of hypertrophic nonobstructive cardiomyopathy (20 yrs), coronary heart disease (10 yrs) and diabetes (5 yrs), presented abdominal pain and discharge of kidney stones when she was 30 years old. Since then, she had experienced similar symptoms attack and calculi discharge more than ten times in the next 20 years, and accepted therapy of extracorporeal shock-wave lithotripsy (ESWL) for five times. At the age of 52, she was admitted into our hospital as a result of oliguria and abdominal pain. Abnormal laboratory findings (BUN: 16.23 mmol/L, Scr: 1044 μmol/L) revealed remarkable renal dysfunction and abdominal Computer Tomography (CT) scan confirmed multiple stones in both her kidneys and the right proximal ureter (Fig. 2A). She accepted hemodialysis (HD) through arteriovenous fistula as renal replacement therapy. A month later, however, the patient failed to improve her renal function after a surgery of right percutaneous pyelostomy, and repeated CT examinations exhibited medullary nephrocalcinosis (Fig. 2B). Two months later, extensive calcification in renal parenchyma and kidney atrophy were observed (Fig. 2C). A year later, she died at home probably caused by discontinuation of dialysis.

For the patient IIe who was the younger brother of the patient IIa, his first episode of “kidney stone” with left abdominal pain and gross hematuria, occurred when he was around 20 years old. In the next more than ten years, he discharged renal calculi for eight times, and accepted ESWL for six times. The first time when he was admitted into our hospital at the age of 38, his laboratory testing (BUN: 7.8 mmol/L, Scr: 210 μmol/L) suggested mild renal impairment. Based on the result of CT which showed multiple high-density stones in both renal pelvis and a high-density stone with a diameter of about 2 cm in the ureteropelvic junction (UPJ) leading to hydronephrosis in the right kidney (Fig. 2D), he underwent double-J ureteral stents insertion following right ureterolithotomy. Transient improvement of renal function (Scr decreased to 149 μmol/L) was observed, however, recurrent ureteral calculus and upper urinary tract infection followed. At the age of 39, he was hospitalized in our blood purification center due to a high Scr level of 998 umol/L. Meanwhile, abdominal CT displayed bilateral kidney atrophy and medullary annular calcium deposition (Fig. 2E). Four months later, patient IIe received allogenic renal transplantation after repeated CT examination demonstrating bilateral diffuse renal calcification (Fig. 2F). The transplanted kidney functioned well at first reflected by significantly reduced Scr level of 149 μmol/L, however, worsening renal function occurred with Scr of 231 μmol/L and 342 μmol/L one and two months after the transplantation, respectively. Linear high-density in the medulla and mild hydronephrosis of transplanted kidney (Fig. 2G) could be seen on the examination of CT scan two months after operation. Isolated kidney transplantation finally failed, with subsequent Scr level of 561 μmol/L and a CT scan result revealing an enlarged transplanted kidney with more evident high-density of renal parenchyma (Fig. 2H). Eventually, patient IIe died of uncontrollable pulmonary infection, of which inevitable immunosuppression might be an important contributor.

Another sibling patient IIf, who was the younger sister of the two probands IIa and IIe, appeared intermittent abdominal pain and urine excretion of stones with a slightly earlier onset at 17 years old. She was once received ESWL and left ureterolithotomy when she was 36 and 37, respectively, with preserved renal function (Scr: 94 μmol/L) at that time. At the age of 39, the abdominal CT scan displayed bilateral renal stones (a larger stone was in the right kidney with a diameter of 1.3 cm) but without nephrocalcinosis (Fig. 2I), and her random urine oxalate-to-creatinine ratio (OCR) elevated abnormally (0.35 mmol/mmol, normal value < 0.04). Of note, a 3-month of supraphysiological dosage of pyridoxine (Vitamin B6, 5 mg/kg·d) prescribed for patient IIf induced a significant reduction more than 30% in random OCR (decreased to 0.20 mmol/mmol, the mean of measurements for three times). Additional medication of hydrochlorothiazide (12.5 mg/d) for a month contributed to decrease of the Ca/Cr from 0.18 to 0.10 (mmol/mmol). On the basis of vitamin B6 and hydrochlorothiazide, patient IIf also received the therapy of sodium citrate and potassium citrate. In the follow-up for more than 1 year, she was always in stable condition without further renal impairment and development of kidney stones.

Evidence of secondary nephrolithiasis history like hyperparathyroidism, renal tubular acidosis (RTA) or Batter syndrome was absent in this family. Recurrent nephrolithiasis and nephrocalcinosis, failure of kidney transplantation or obviously elevated OCR, any of above abnormalities highly suggested a diagnosis of PH1^10^. According to pedigree analysis, there were 3 diseased siblings (two females and one male) in one generation, whose phenotypes conformed to the autosomal recessive inherited disease. None of the remaining family members presented any sign of renal calculus.

### Mutation analysis of *AGXT*

**Figure 1:**
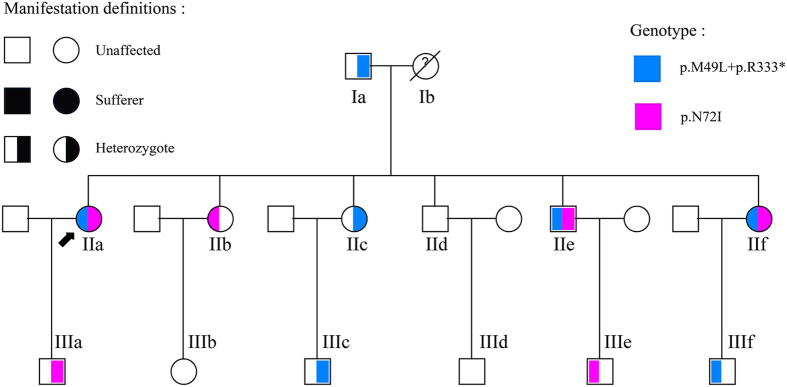
Pedigree of the Chinese family with Primary hyperoxaluria type 1. □, male; ○, female; ↗, proband.

**Figure 2:**
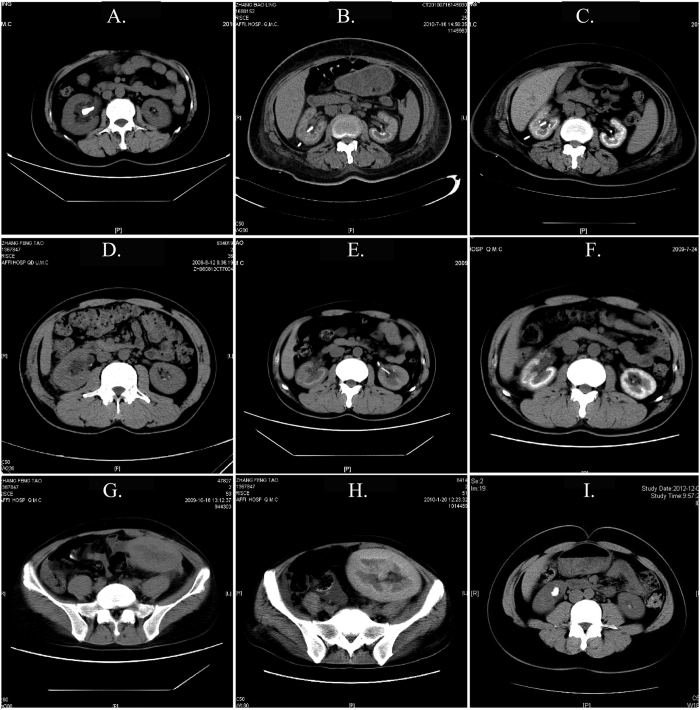
CT scanning at different stages of three Chinese siblings of IIa, IIe and IIf with Primary Hyperoxaluria Type 1. (**A**–**C)** CT scanning of bilateral kidneys of proband IIa; (**D**–**H)** CT scanning of bilateral kidneys of sibling IIe; (**I)** CT scanning of bilateral kidneys of sibling IIf.

**Figure 3:**
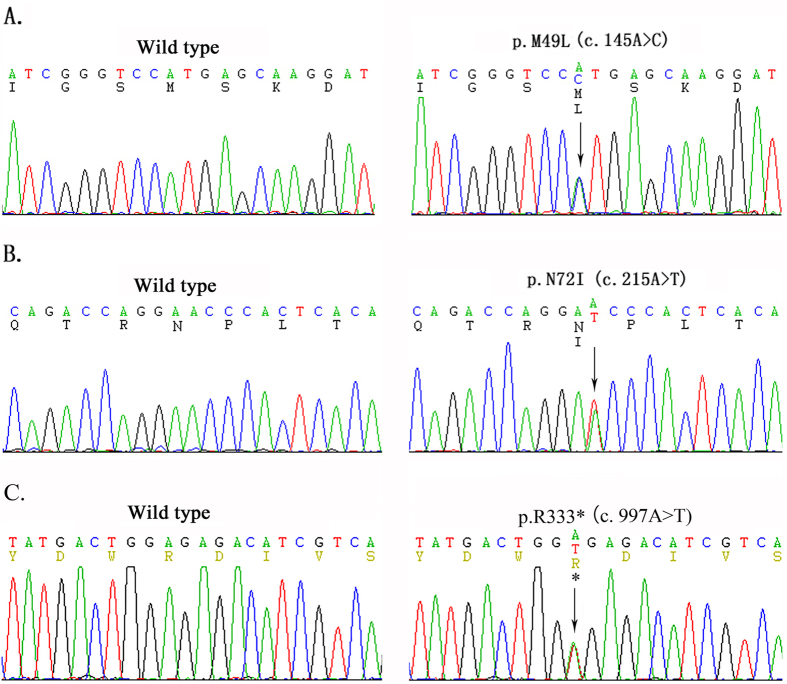
Three AGXT mutations identified in the Chinese family with Primary Hyperoxaluria Type 1. (**A**) wild type(left panel) and heterozygous mutant type (right panel) of p.M49L; (**B**) wild type(left panel) and heterozygous mutant type (right panel) of p.N72I; (**C**) wild type(left panel) and heterozygous mutant type (right panel) of p.R333*. Mutation naming and description rules refer to the latest guideline published by Human Genome Variation Society (http://www.hgvs.org/mutnomen/recs.html). *AGXT*-mRNA (NM_000030) starts from the first base of the first codon.

In this study, two novel missense mutations which were p.M49L (c.145A > C) and p.N72I (c.215A > T) and a previously reported nonsense mutation p.R333* were detected by direct sequencing in all family members except Ib who was dead (Figs 1 and 3). Moreover, analysis of three common polymorphisms, P11L, I340M and IVSI+74 bp, revealed that only IVSI+74 bp was found in this family. Missense mutation p.M49L was in cis configuration with nonsense mutation p.R333* in the minor allele characterized by IVSI+74 bp, while the other missense mutation p.N72I was in trans with both p.R333^*^ and P.M49L in the major allele in which none of three common polymorphisms was detected. In this study, we called the mutated minor allele “AGT-Mi” and the mutated major one “AGT-Ma”, and the normal major allele “AGT-MA”. As we can see from Fig. 1, three clinically confirmed patients IIa, IIe and IIf were all *AGXT* compound heterozygotes with the same genotype (AGT-Mi and AGT-Ma); seven healthy family members were also identified as heterozygotes (Ia, IIc, IIIc and IIIf carried AGT-Mi and AGT-MA, while IIb, IIIa and IIIe carried AGT-Ma and AGT-MA). The rest of nine members of this family were genetically screened unaffected by any *AGXT* mutation.

Three web based programs were used to predict effects of the two missense variants, and it turned out that PolyPhen-2 and SIFT predicted p.M49L as a benign mutation, Mutation Taster showed it was disease-causing (with probability scores of 0.999998505384457), however. Meanwhile p.N72I was considered as a destructive mutation by all of these three established online tools. To our knowledge, p.N72I has never been reported to databases before; according to the ExAC database, the p.M49L variant has been observed in 53 individuals in the heterozygous state with an allele frequency of 0.0004. Both p.M49L and p.N72I were highly conserved in all 8 species of homologous proteins (Supplemental Figure 1). In addition, Grantham Matrix score system estimating pathogenicity showed a result that p.M49L was moderately pathogenic whereas p.N72I scored high (Table 2). The 3D Model and the positions of both mutations p.M49 and p.N72 in it are shown in Supplemental Figure 2.

Direct sequencing analysis failed to find above-mentioned two mutations in 100 healthy subjects from the same ethnic origin, in contrast, the minor allele frequency (MAF) of IVSI+74 bp, P11L and I340M in the control group was 3%, 2% and 8%, respectively. Information about the MAF of those above three polymorphisms in four publicly available databases (ExAC, 1000 Genome, GO-ESP and AGXT mutation database^19^) was shown in Supplemental Table 1.

### Morphological and structural difference in stones

**Figure 4:**
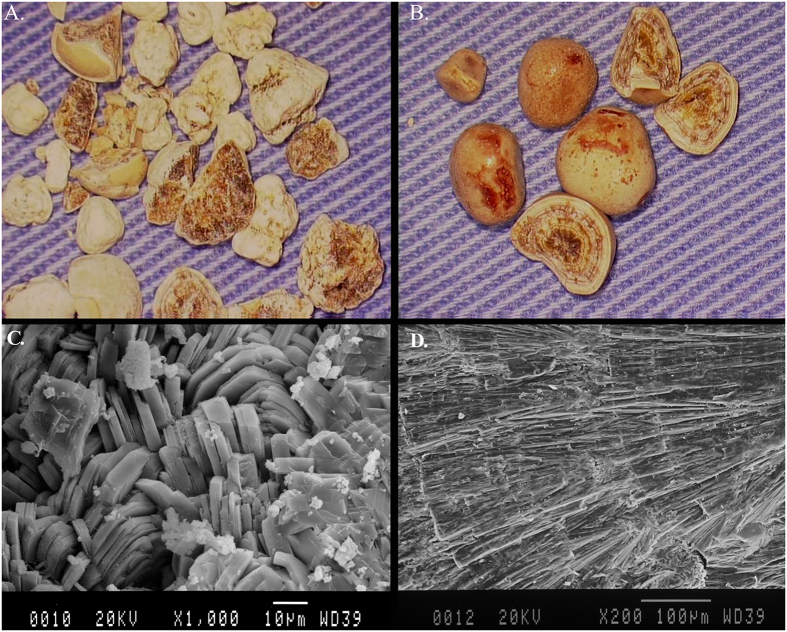
Morphologic characteristics of Calcium Oxalate Monohydrate stones from two Chinese siblings with Primary Hyperoxaluria Type 1. Panels (A,B) show stone surfaces on stereomicroscopic examination (×10), and Panels (C,D) show the sections on scanning electron microscopy. The stones of both siblings had light-brown surfaces. Stones from sibling IIa showed irregular in shape, ununiformity in size, and inhomogeneous internal structures with the aggregations of various sizes and dimensions plate-like crystals (**A**,**C**); In contrast, the stones from IIf had smooth surface, regular shape, well-organized geometric cross sections with more compact and radiating inner structure (**B**,**D**).

An early research demonstrated that stones from patients with PH1 are lightyellow in color, irregular in shape and loose in section appearance with crystal aggregation in various sizes, however, stones produced by those with idiopathic hyperoxaluria show puce and regular looks with dense, radiating charcoal-like sections^16^. In our investigation, the calculi, that we had the opportunity to have from both patient IIa and her sibling IIf, had a similarly high calcium oxalate monohydrate (COM) content (>95%) to idiopathic calcium stones, and a similar light-brown color surface. However, other distinctly different morphologic characteristics between them could be seen. Stones from patient IIa showed irregular in shape and ununiformity in size on stereomicroscopic examination, and scanning electron microscopy displayed the inhomogeneous internal structures with the aggregations of various sizes and dimensions plate-like crystals (Fig. 4A,C). These features were close to the description for PH1. In contrast, the stones from IIf demonstrated a smooth surface, a regular shape and a well-organized geometric cross section (Fig. 4B). Under the examination of scanning electron microscopy, the stones exhibited a more compact and radiating inner structure with fine-grained concentric laminations (about 100 μm thick) (Fig. 4D). These features of stones from IIf were in accordance with characteristics of idiopathic COM stones^20^, but the latter are commonly darker in appearance.

## Discussion

To date, there are more than 190 known disease-causing mutations in *AGXT* and of these 120 are missense mutations. The remainders are null mutations (such as deletion/insertion, nonsense and splice site mutations) which probably produce truncated AGT protein with definite pathogenicity^1^. Nevertheless, it needs considerable work to assess and confirm whether a missense mutation could induce defects of corresponding protein. In our investigation, all three patients were at compound heterozygous state and seven carriers were not diseased, which showed co- segregation of the mutant alleles with PH1 under an autosomal recessive pattern. The nonsense mutation p.R333* was recognized as a devastating change that could affect synthesis of AGT definitely. In general, p.M49L might be considered as a benign variant given that it occurred in the same minor allele with a nonsense mutation (p.R333*). However, prediction based on online software, sequence alignment, and control population from this study or databases displayed that p.N72I showed strong possibility of causing disease, while p.M49L was moderately pathogenic. Therefore, it deserves further analysis and investigation to confirm whether p.M49L is a “real” PH1 mutation, or a nonfunctional variant, or an alteration with the similar potential effect as the polymorphisms of P11L or I340M.

The solved crystal structure of AGT reveals that this compact dimer with one PLP-binding site in each subunit is a member of Fold Type I family of PLP-enzymes^21^. Each subunit consists of a long unstructured N-terminal tail (residues 1–21) that wraps over the surface of the other subunit, a large domain (resides 22–282) containing most of the active site and dimerization interface, and a small domain (residues 283–392) which expresses the peroxisome targeting information^3^,^22^. The cofactor, PLP, is covalently bound to the apoprotein with a lysyl residue at the position 209^3^. In the N-terminal tail, the minor allele P11L which is not itself disease-causing has been proved to interact synergistically with some mutations to cause PH1, facilitating mislocalizing AGT from peroxisome to mitochondria by generating a mitochondria targeting sequence (MTS)^23^,^24^. Besides, P11L is also responsible for unfolding, instability or catalytic activity reduction of AGT when appears with some certain mutations^2^,^5^,^22^,^25^. In this family, however, only minor allele characterized by IVSI+74 bp was detected but P11L and I340M were not. Furthermore, IVSI+74 bp doesn’t act like P11L do to take part in the disease process, highlighting that it was the two novel mutations what should be concentrated on to explore the mechanism of PH1^26^,^27^.

Through 3-D structure analysis of AGT homodimer, we learned that p.M49L was located in the interface connecting two monomers, while p.N72I was at the outside surface of spatial structure of AGT (Supplemental Figure 2). Neither p.M49L nor p.N72I was sited in any catalytic site, PLP-binding site or acknowledged peroxisome targeting sequence. A previously found mutation p.G47R was reported as a detrimental mutation under the background of minor allele (G47R-Mi) by the following mechanisms of altered subcellular localization, folding defect, aggregation and proteolytic degradation. In addition, similarly to G47R-Mi, G41R-Mi was also featured by mistargeting and a remarkable propensity to proteolytic degradation and aggregation^5^,^28^. Thus, we could speculate that the novel missense mutation p.M49L which was close to p.G47R and p.G41R not only in the primary structure but also in the spatial structure (Supplemental Figure 2A), would probably influence AGT by the same or similar mechanisms. Intriguingly, different responsiveness to pyridoxine between G41R-Mi and G47R-Mi was noted, which was that pyridoxine played a positive role in folding or dimerization of G47R-Mi, while it was found to have little or no effect on G41R-Mi^22^,^29^. We can learn from AGT crystal structure that Gly41 lies in a α-helix, whereas Gly47 lies in a mobile loop, however, it seems that it is far from enough to predict pathogenicity based only on the structure context. Therefore, exploring the potential molecular mechanism of p.M49L in PH1 by function research will be particularly intriguing. Regarding the missense mutation p.N72I, its pathogenicity could be partly conjectured according to the p.T70N, which was the closest mutation to p.N72I so far and was proved deleterious by liver biopsy (Williams and Rumsby unpublished data^19^). Moreover, additional two residue positions, Met195 and Gly253, were observed also adjacent to Asn72 in the crystal structure (Supplemental Figure 2B). A previous rigorous study showed that variants of both Met195 (M195R, M195L) and Gly253 (G253R) could cause defects in activity or stability of AGT under the background of either minor allele or major allele^23^. Hence, p.N72I and p.R333* from two separate alleles should be the dominant mutations in this family, however, the potential pathogenic effects of p.M49L, that has been revealed by above-mentioned in-depth analyses, could not be ignored.

Apparently, either in four publicly available databases or in reports with regard to European and North American populations^30^, the MAF of three common *AGXT* polymorphisms including P11L, I340M and IVSI+74 bp were all significantly higher than that of Chinese population in this study. This difference may demonstrate distinct genotypic discrepancy among different geographical regions and different ethnic groups. Considering the weak negative effect of these polymorphisms on AGT, and their synergetic effect with some missense mutations, the question of whether the lower MAF in this population is associated with a decreased prevalence of PH1 asks for further investigation to answer.

It is well known that genotypic heterogeneity and expression variability exist in PH1, which requires physicians to make more efforts to differentiate diverse PH1 forms. Five major initial presentations have been summarized in the literature: (1) the infantile form with early nephrocalcinosis and kidney failure; (2) childhood or adolescence presentation with recurrent urolithiasis and progressive renal failure; (3) the adult form with occasional renal stones; (4) the post kidney transplant form; (5) the presymptomatic form with family history^31^. In this present work, we described two sibling PH1 patients (IIa and IIe) who belonged to the adult form and one patient (IIf) in the same generation whose first manifestation appeared at late adolescence. Despite all of three patients were found with identical compound heterozygous *AGXT* mutations, patient IIa and IIe underwent more serious clinical courses and more accelerated progression to ESRD compared with patient IIf who was still with preserved renal function, reflecting a certain extent of phenotypic heterogeneity. Yet continued follow-up should be implemented on patient IIf to evaluate her disease evolution.

A number of data show that the final outcome of PH1 benefits much from presymptomatic screening and timely accurate diagnosis which mainly depends on *AGXT* mutation screening, liver biopsy, or elevated plasma and urine oxalate level and so on. Sometimes, however, misdiagnosis or missed diagnosis of PH1 can happen when no rapid and reliable examination is available. Therefore, more PH1-specific information should be collected and applied to promote correct diagnosis.

It has been reported that specific analysis of composition, shape, color, texture and internal microstructure of kidney stones from PH1 patient, is of great value for earlier definitive diagnosis^16^. Interestingly, in this study, although highly similar in composition and surface color, there was distinct difference in shape and internal structure of stones obtained from two PH1 siblings who shared the same genotype, suggesting some distinction in the mechanism of calculus formation and development between the two patients. Such conclusion is not surprising or unexpected since in an absence of considerable mechanical force capable of modelling the stone external shape, as in the kidney, nothing else than the mechanism of development can determine the outward calculus shape. Absence of organized structure in the stone body of patient IIa, indicated the very rapid and on-going crystal formation. However, the regularly structured layer consisting of tiny crystals in calculus of sibling IIf, might reflect a slow (This implies that the stone was in a prolonged contact with a supersaturated urine.) and intermittent (probably caused by the balance shift of between preventive and promotive mechanisms) growth rate. A faster growth rate is probably related to higher levels of oxalate excretion in urine, accordingly, we suppose that different dietary habits might play an important role in the morphology and structure diversity of kidney stones from the two siblings since they had the same genotype. Nevertheless, the local environment conditions, such as different locations of stone formation, various bacterial infections caused by urinary tract obstruction^32^, and so on, might also be significant determining factors. On the other hand, it’s worth noting that our findings, PH1 stones occasionally mimicking the common type of whewellite stone in morphology, further enrich phenotype diversities of PH1, and remind urologists and nephrologists to think of the possibility of this disease when met stones like this so as to avoid missed or delayed diagnosis.

Pyridoxine as an essential cofactor of AGT, substantial evidence had proved that approximately 30% of PH1 patients reveal responsiveness to pyridoxine in their therapy. The most representative example is that a majority of the homozygotes of p.G170R associated with AGT mis-targeting to mitochondria display complete sensitivity to pyridoxine through rescuing enzyme mis-targeting and stabilizing the dimer^23^,^33^. Pyridoxine therapy is also used in other diseases affecting pyridoxal-5′-phosphate-dependent enzymes^34^,^35^. Its mechanisms of action are not entirely elucidated but include both a prosthetic group and a chaperone effect. This study demonstrated that pyridoxine suggested some efficacy for patient IIf, however the potential molecular mechanisms of this partial treatment effectiveness to this genotype of PH1 remain unclear and deserve further investigation to elucidate.

Although many conservative treatments including pyridoxine have showed certain ability to relieve clinical symptoms and slow progression to ESRD^36^, the final therapy to cure PH1, however, would rely on preemptive liver transplantation or combined liver-kidney transplantation. The patient IIe, whose diagnosis of PH1 was not made until after death, suffered a rapid failed isolated kidney transplantation which might due to huge amount of excretion of oxalate accumulation in his body. This tragedy reminded us once again the importance of establishing the diagnosis of PH1 before transplantation, and a great necessity of taking steps to decrease the oxalate load prior to surgery to prevent damage to the new kidney^37^.

In conclusion, two novel *AGXT* missense mutations (p.M49L and p.N72I), which will enrich the *AGXT* mutation database and provide a better comprehension of PH1 pathogenesis, were identified in a big Chinese PH1 family. Significant morphological and structural difference of kidney stones from two siblings with the same genotype observed in this study displays the heterogeneity of genotype-phenotype correlation from a different angle, adds more information to understand the factors involved in the stone formation and development, and facilitates to differentiate PH1 from other kinds of nephrolithiasis.

## Subjects and Methods

### Diagnostic criteria

Subjects were selected according to the diagnostic standard for PH1 in adults: bilateral nephrocalcinosis or recurrent urolithiasis, with or without complication of renal dysfunction, accompanied with one of following conditions: (1) biochemistry parameters: random oxalate-to-creatinine ratio (OCR) is more than 0.04 (mol/mol), elevated urine hydroxyacetic acid; (2) Enzymology: loss of catalytic activity of AGT in liver tissue (2–48% of remaining activity can be seen in 30–50% of survivals, requiring other combined biochemistry tests or molecular/gene diagnosis); (3) gene mutations: both allelic genes of AGXT include pathogenic mutations.

### Patients

The subjects were a large Chinese family from Shandong province, China. After written informed consent was obtained, blood samples were collected from all family members. The study was approved by the Ethics Committee of the Affiliated Hospital of Qingdao University and the methods were carried out in accordance with the approved guidelines.

### Mutation analysis of *AGXT*

Genomic DNA was extracted from the peripheral blood of the patients and their family members by GenElute blood genomic DNA Kit (Sigma, NA2010). The primers (Sangon Biotech, Shanghai) of *AGXT* designed by Monico, *et al*.^38^ were generated to amplify all exons and flanking intronic regions of *AGXT* which contains 3 common polymorphisms involving P11L, I340M and IVSI+74 bp. PCR was performed to amplify all above fragments using Taq polymerase (Takara EX Taq Hot start version, DRRR006B) according to the manufacturer’s instructions. PCR samples were subjected to bidirectional sequencing running on an ABI Prism 3700 DNA Analyzer (Applied Biosystems, Calif, USA). 100 unrelated healthy subjects with age range to 30 to 67 were selected as a control group. None of them had past history of kidney stone. We adopted direct sequencing to verify mutations found in our investigation and to analyze the MAF. Sequence analysis and sequence alignment were underwent by *in silico* software Chromas 2.31 and Vector NTI Advance 11.5.

Three online programs like PolyPhen-2(http://genetics.bwh.harvard.edu/pph2)/, SIFT (http://sift.jcvi.org/www/SIFT_enst_submit.html) and Mutation Taster (http://www.mutationtaster.org/) were employed to predict pathogenicity of putative missense mutations. On the basis of matrix algorithm described by Grantham^39^ and Abkevich^40^, further score system evaluating the deleteriousness of missense mutation was used in this study. Then we performed sequence alignment on 8 species of AGT homologous proteins through Vector NTI Advance 10-Align. Those eight species of proteins were as follows: Homo sapiens (NP_000021.1), Canisnis familiaris (XP_848328.1), felis catus (CAA53527.1), Oryctolagus cuniculus (P31030.1), Rattus rattus (CAA29656.1), Mus musculus 1(AAH25799.1), Xenopus tropicalis (NP001006705.1) and Danio rerio (AAH67638.1). The 3D structural analysis of AGT was performed by Cn3D-4.3.1.

### Analysis of urinary calculi

We observed and studied apparence and section structure of stones available from patient IIa, and patient IIf by stereomicroscopic examination and scanning electron microscopy. Compositional analyses of stones were performed by fourier transform infrared spectrometer (FTIR).

## Additional Information

**How to cite this article**: Wang, C. *et al*. Two novel *AGXT* mutations identified in primary hyperoxaluria type-1 and distinct morphological and structural difference in kidney stones. *Sci. Rep.* **6**, 33652; doi: 10.1038/srep33652 (2016).

## Supplementary Material

Supplementary Information

## Figures and Tables

**Table 1:** Manifestations and biochemical features of three patients in family with PH1.

Item	IIa^a^	IIe^a^	IIf^b^
Gender	female	Male	Female
Age (yrs)	52	38	37
Height(cm)^c^	168(159)	178(170)	172(159)
BMI(kg/m^2^)	21.26	23.04	22.0
Blood PH	NA	NA	7.4
K^+^(mmol/L)	5.3	4.8	4.0
Cl^−^(mmol/L)	108	107	101
CO_2_CP(mmol/L)	18.2	18.6	24.7
Ca^2+^(mmol/L)	2.38	2.30	2.42
Scr(μmol/L)	1044	210	94
eGFR(ml/min)^d^	3.6	32.8	62.0
Urine pH	6.0	6.5	6.0
OCR	NA	NA	0.35
Nephrolithiasis	Yes	Yes	Yes
Nephrocalcinosis	Yes	No	No

eGFR Estimated glomerular filtration rate; NA Not available; OCR random oxalate-to-creatinine ratio (normal level in adults ≤0.04).

^a^Information of patients when they was admitted to Affiliated Hospital of Qingdao University for the first time.

^b^Information of patients when they was in follow-up for the first time.

^c^Average heights of Chinese adult males, females or people at corresponding ages.

^d^Calculated by MDRD formula.

**Table 2:** Pathogenic scoring of *AGXT* missense mutations.

Gene mutation	Protein mutation	Grantham Matrix Scoring^[11−12]^	MSA Scoring	Total Score
c.145A > C	p.M49L	15(1)	8/8(5)	6
c.215A > T	p.N72I	149(4)	8/8(5)	9

Grantham Matrix scoring: <60.0 = 1 point, 60.0–78.3 = 2 points, 78.4–93.4 = 3 points, >93.4 = 4 points, any substitution of cysteine = 5 points. MSA scoring: conservative in all eight of species = 5 points, 6–7 species = 4 points, 4–5 species = 3 points, 1–3 species = 1 point. Total score: ≥8 high pathogenicity, 6–8 moderate pathogenicity, ≤5 probably nonpathogenic (like polymorphism). MSA: multiple sequence alignment.
