# Supplementary material for: Two novel AGXT mutations identified in primary hyperoxaluria type-1 and distinct morphological and structural difference in kidney stones
Source: Sci Rep. 2016 Sep 20;6:33652. doi: 10.1038/srep33652 (PMC5028881; doi:10.1038/srep33652)
Supplement: Supplementary Information [file srep33652-s1.pdf]

TITLE PAGE

Title: Two novel *AGXT* mutations identified in primary hyperoxaluria type-1 and distinct morphological and structural difference in kidney stones

Cui Wang[1,2], Jingru Lu[1,2+], Yanhua Lang[1+], Ting Liu[1,2+], Xiaoling Wang[1,2+], Xiangzhong Zhao[2*], Leping Shao[1,2*]

[1]Department of Nephrology, [2]Central Laboratory, the Affiliated Hospital of Qingdao University, 16 Jiangsu Road, Qingdao 266003, China.

[*]Corresponding Author:

Leping Shao, Central Laboratory and Department of Nephrology， the Affiliated Hospital of Qingdao University, 16 Jiangsu Road, Qingdao 266003, China. Tel: +8653282911301; Fax: +8653282911093; E-mail: lepingshao@163.com

And

Xiangzhong Zhao, Central Laboratory, the Affiliated Hospital of Qingdao University 1677 Wutaishan Road, Qingdao 266555, China. Tel: +8653282917303; Fax: 8653282917303; E-mail: fzzg.2002@163.com

[+] Theses authors contributed equally to this work.

Competing Interests: The authors have declared that no competing interests exist.

Word count: Abstract 194 words; Text 4500 words.

Supplemental Figures

Supplemental Figure 1

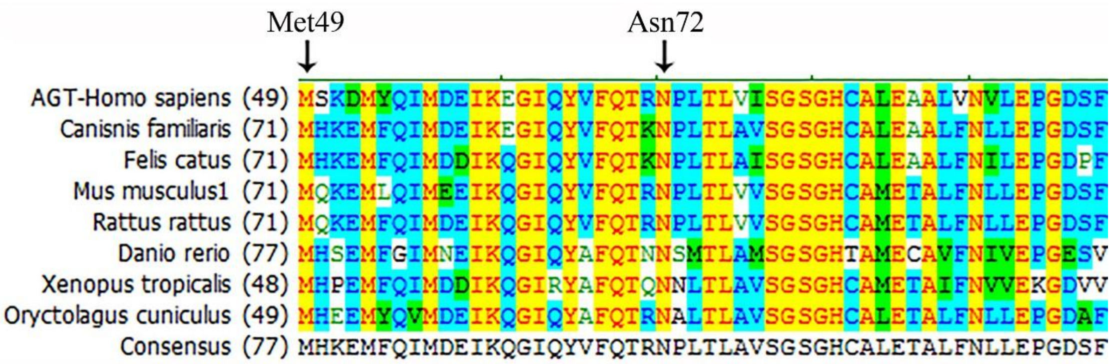

Annotation: The result of sequence alignment on 8 species of AGT homologous proteins. Black arrows are pointing to methionine at position 49 and asparaginate at position 72 in Human AGT, respectively.

Supplemental Figure 2

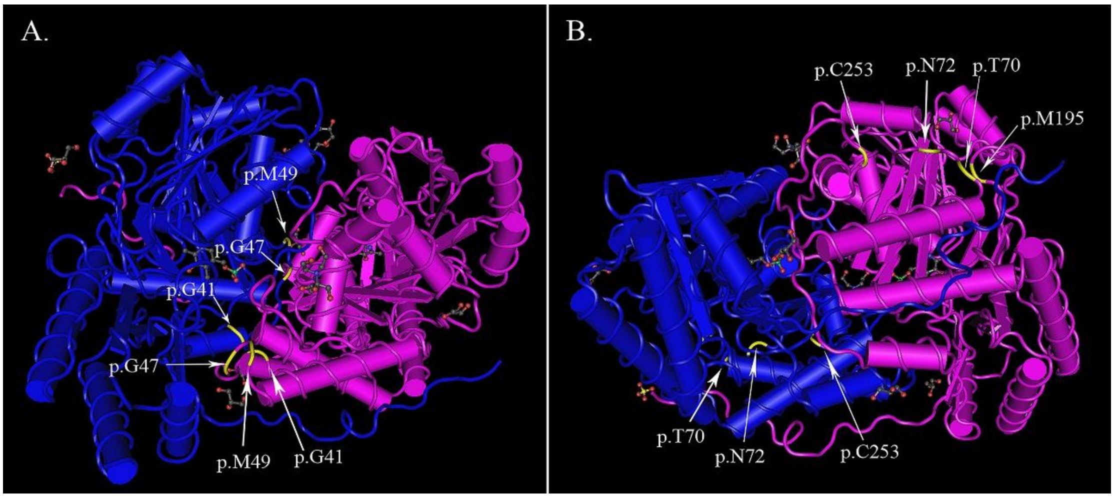

Annotation: AGT 3D structure (Cn3D-4.3.1) and positions of Met49 and Asn72 residues in it. The figure reprensents the crystal structure of AGT with one monomer blue and the other red. A. Gly41, Gly47 and Met49 residues (marked with yellow and arrows) locate in the interface of AGT dimer; B. Thr70, Asn72, Met195 and Cys253 residues (marked with yellow and arrows) lie in outside surface of AGT.

Supplemental Table

Supplemental Table 1 Minor allele frequency (MAF) of three minor in four publicly available databases and this study

			Frequency in controls				
Sequence variant	Condon/effect	SNP database	ExAC	1000 Genome	GO-ESP	AGXT mutation database	This study
c.165+19_165+92dup74		rs180177174	NA	NA	NA	0.24	0.03
c.32C>T	p.P11L	rs34116584	0.1545	0.0809	0.1533	0.20	0.02
c.1020A>G	p.I340M	rs4426527	0.1653	0.1112	0.1592	0.15	0.08

NA: not available
